# Patient’s Healthy-Limb Motion Characteristic-Based Assist-As-Needed Control Strategy for Upper-Limb Rehabilitation Robots

**DOI:** 10.3390/s24072082

**Published:** 2024-03-25

**Authors:** Bingjing Guo, Zhenzhu Li, Mingxiang Huang, Xiangpan Li, Jianhai Han

**Affiliations:** 1 School of Mechatronics Engineering, Henan University of Science and Technology, Luoyang 471003, China; 220320010009@stu.haust.edu.cn (Z.L.); huangmingxiang@163.com (M.H.); xiangpanli@haust.edu.cn (X.L.); jianhaihan@haust.edu.cn (J.H.); 2 Collaborative Innovation Center of Henan Province for High-End Bearing, Luoyang 471003, China; 3 Collaborative Innovation Center of Machinery Equipment Advanced Manufacturing of Henan Province, Luoyang 471000, China

**Keywords:** upper-limb rehabilitation robot, assist-as-needed control, endpoint stiffness estimation, stiffness mapping algorithm, surface electromyographic signal

## Abstract

The implementation of a progressive rehabilitation training model to promote patients’ motivation efforts can greatly restore damaged central nervous system function in patients. Patients’ active engagement can be effectively stimulated by assist-as-needed (AAN) robot rehabilitation training. However, its application in robotic therapy has been hindered by a simple determination method of robot-assisted torque which focuses on the evaluation of only the affected limb’s movement ability. Moreover, the expected effect of assistance depends on the designer and deviates from the patient’s expectations, and its applicability to different patients is deficient. In this study, we propose a control method with personalized treatment features based on the idea of estimating and mapping the stiffness of the patient’s healthy limb. This control method comprises an interactive control module in the task-oriented space based on the quantitative evaluation of motion needs and an inner-loop position control module for the pneumatic swing cylinder in the joint space. An upper-limb endpoint stiffness estimation model was constructed, and a parameter identification algorithm was designed. The upper limb endpoint stiffness which characterizes the patient’s ability to complete training movements was obtained by collecting surface electromyographic (sEMG) signals and human–robot interaction forces during patient movement. Then, the motor needs of the affected limb when completing the same movement were quantified based on the performance of the healthy limb. A stiffness-mapping algorithm was designed to dynamically adjust the rehabilitation training trajectory and auxiliary force of the robot based on the actual movement ability of the affected limb, achieving AAN control. Experimental studies were conducted on a self-developed pneumatic upper limb rehabilitation robot, and the results showed that the proposed AAN control method could effectively estimate the patient’s movement needs and achieve progressive rehabilitation training. This rehabilitation training robot that simulates the movement characteristics of the patient’s healthy limb drives the affected limb, making the intensity of the rehabilitation training task more in line with the patient’s pre-morbid limb-use habits and also beneficial for the consistency of bilateral limb movements.

## 1. Introduction

The process of aging, degeneration, and disease-related damage in the central nervous system typically results in motor dysfunction, significantly affecting the daily activities of patients [1,2]. To restore all or part of their motor functions, patients need to receive prolonged rehabilitation therapy to induce neuroplasticity [3]. Achieving brain neuroplasticity from conventional rehabilitation therapy involves the coordination of multiple therapists, which makes it a labor-intensive and long-term process. Therefore, manual rehabilitation therapy presents limitations, including resource inadequacy, high financial investments, variability in training quality, and therapist burnout. To solve these issues, the development of robot-assisted therapy is emerging as a promising avenue for rehabilitation treatment. This approach offers highly repetitive, intensive, adaptable, and quantifiable rehabilitation therapies [4,5].

Recent studies in robotic therapy indicated that the implementation of a progressive rehabilitation training model to promote patients’ motivation efforts can greatly restore damaged central nervous system function in patients [6]. Patients’ active engagement is considered one of the key factors contributing to neural plasticity and motor recovery during the course of therapy. Consequently, researchers proposed the assist-as-needed (AAN) control strategy to better motivate patients’ active voluntary participation in upper limb rehabilitation therapy. The AAN strategy focuses on providing the minimal amount of robotic assistance necessary for a patient to complete a rehabilitation task, while a significant effort is required from the patient [7]. Deploying robotic assistance in accordance with the AAN strategy comes with some technical challenges. These challenges primarily involve effectively assessing patients’ functional capabilities, accurately estimating the required motor support based on patients’ specific disability level or recovery progress, and dynamically adjusting the level of robot assistance in real time. These technical concerns are particularly complex in upper limb rehabilitation training, considering the diverse forms of exercises involved in training for various activities of daily living (ADL) carried out through occupational therapy (OT) for active rehabilitation training.

The assessment of patients’ function abilities is predominantly performed using two main approaches: the biomechanical model-based method [8,9] and the motor performance-based method [7,10,11,12,13]. In the context of biomechanical modeling, a skeletal muscle model is usually constructed and analyzed based on biomechanical theories. This modeling process involves the identification of model parameters to quantitatively evaluate muscle forces and joint torques, thus contributing to the assessment of physical motor ability. For instance, Li Zhijun et al. [9] developed a reference musculoskeletal model of the human forearm’s joints. This model, driven by surface electromyography (sEMG), was utilized to calculate net torque and joint stiffness to match the operator’s motion behavior. Alternatively, a common approach for implementing the AAN strategy is through the utilization of the motor performance-based method. This method involves inferring patients’ assistance requirements based on their performance and then using this information to adapt the level of robotic assistance. Motion performance-based assessment methods can be broadly categorized into two main groups. The first category relies on physical sensors to capture various signals, including commonly measured parameters such as joint position, velocity, and human–robot interaction forces. These signals are utilized to develop empirical formulas for the evaluation of motion performance or establish assessment criteria based on clinical medical scales. In [7], a new functional ability index (FAI) estimation algorithm in accordance with the employed clinical procedure was proposed for the estimation of a subject’s motor ability in a movement task. The FAI evaluation algorithm was obtained by parameter equations including task completion time and angular position and velocity of the upper-limb joints. The position and velocity parameters were determined by the inertial measurement unit (IMU). Pehlivan et al. [10] applied a Kalman filter in conjunction with Lyapunov analysis to estimate the functional capabilities of subjects wearing the RiceWrist-S exoskeleton. The force estimator independently determined the subjects’ capability at each moment in time only based on position detection. The second category of motion performance assessment methods is centered on biological signals, such as electromyographic signals. These signals are harnessed to establish relationships that map to joint moments, facilitating the evaluation of an individual’s physical exercise capacity. Tatsuya Teramae et al. [11] proposed, using EMG signals, to estimate a subject’s torque output. The relationship between the 16 EMG RMS values and the joint torque vector was modeled as a linear torque estimation model, with a neurofuzzy muscle-model matrix modifier. The neurofuzzy modifier outputs the coefficient for each weight of the muscle-model matrix to modify the weight matrix in real time based on the upper-limb posture of the user. This approach establishes a mapping relationship between EMG signals and joint torques through artificial intelligence algorithms such as support vector machines or neural networks. However, these methodologies do not delve into the intricacies of human biomechanical processes and fail to analyze the contributions of different muscles to various motions. 

Building upon the techniques employed to assess a patient’s motor functional ability, the determination of actual assistive torque and the specific implementation method of real-time control have emerged as focal points of research in AAN control. These research areas encompass several approaches, such as direct adjustment of assistive force/torque through force control methods [8,9,12], adaptive adjustment of impedance/admittance coefficients to achieve force/position interaction performance [7,13], and intelligent learning algorithms [14,15]. Shawgi Younis et al. [7] applied an adaptive inertia-related torque controller. This control strategy involves the design of an inner position loop nested within the outer torque feedback loop. The desired torque is computed based on the stiffness decay algorithm integrated with the FAI value, which either strengthens or relaxes the controller’s stiffness to enable the modulation of the assistive torque. Carmichael et al. [8] developed an admittance control scheme to implement the AAN paradigm in the robot. The hand strength of a patient in the task space was calculated by an upper-limb musculoskeletal model. A task model calculated the strength required for the ongoing task. This calculated task’s strength requirement was then compared with the operator’s strength capability to gauge the assistance force which was the input of the admittance controller. In [12], an upper limb mirror control strategy based on an adaptive AAN approach is proposed. This adaptive AAN module combines the traditional impedance control with a method for assessing the movement state of the affected limb. It automatically adjusts the auxiliary force applied to the affected limb in real time to maximize the active torque of the affected limb. WANG et al. [13] presented an AAN control strategy for wrist rehabilitation robots. In this work, specific rules for evaluating patients’ abilities were established, and patients’ functional capabilities were assessed in accordance with these predefined criteria. The controller was designed based on the impedance control theory and a dynamics model. It dynamically adjusted the impedance coefficients in response to both the reference trajectory and the assessment of the affected limb’s kinematic ability, enabling the precise and on-demand modulation of the total output torque of the robot.

With the rapid development of artificial intelligence, researchers have increasingly focused on intelligent control methods with inherent learning capabilities for upper-limb rehabilitation exoskeleton robots, for example, neural network control methods, reinforcement learning control methods, etc. In [14], a greedy AAN (GAAN) controller was designed for the upper limb rehabilitation training of neurologically impaired subjects. The GAAN control paradigm includes a baseline controller and a Gaussian radial basis function (RBF) network. This RBF network plays a pivotal role in modeling the functional capabilities of the subjects. The weight vectors of RBF networks evaluating the subjects’ impairment level are updated according to a greedy strategy, so that the maximum force provided by the subjects is gradually learned over time.

The aforementioned forms of adaptive AAN control primarily focus on assessing in a patient the motor functional ability of the affected limb. In the process of formulating task-specific assistive guidelines, the requisite torque for task completion has traditionally been ascertained through either modeling the rehabilitation task or integrating interactive torques of the affected limb into the robot’s dynamic model. These methods are subjective and experience-based, representing a form of artificially set expectations. As a result, AAN control, in which the designer determines the desired outcome, tends to diverge from patients’ expectations and is less adaptable to the diverse requirements of individual patients.

In view of the above problems, this paper introduces a novel AAN control method based on the motor performance of the patients’ healthy limb. The study was conducted on a self-developed pneumatic upper-limb rehabilitation robot. Employing the AAN control strategy, this system facilitates the performance of collaborative rehabilitation training tasks by the patient and the robot. It achieves this by leveraging both the active torque generated by the patient and the torque output from the robotic system. To implement the AAN control, myoelectric signals and human–robot interaction forces are collected during patients’ movement to assess their motor ability. This assessment involves a comparison with and the analysis of the motor performance of the healthy limb to accurately identify deficiencies in the affected limb motor abilities when performing the same movements. Subsequently, the robot’s rehabilitation training trajectory and assisting force are dynamically adjusted based on the actual motor performance of the affected limb. The significance of this study lies in utilizing the patient’s healthy limb performance as a benchmark, comparing it with the affected limb’s motor capabilities to determine the robot’s assisting torque. Additionally, the stiffness information of the healthy limb is utilized as a criterion to determine the necessary motor ability to complete the task, as stiffness reflects the force/position interaction characteristics of the human body during the rehabilitation process. This method makes robot-assisted rehabilitation training more in line with patients’ own characteristics and hand habits. Moreover, the rehabilitation robot’s motions adjusted in time according to the flexibility of the human healthy limb are more natural. These refinements significantly enhance the comfort experienced by the affected limb.

## 2. Upper-Limb Rehabilitation End-Effector Robot

**Figure 1:**
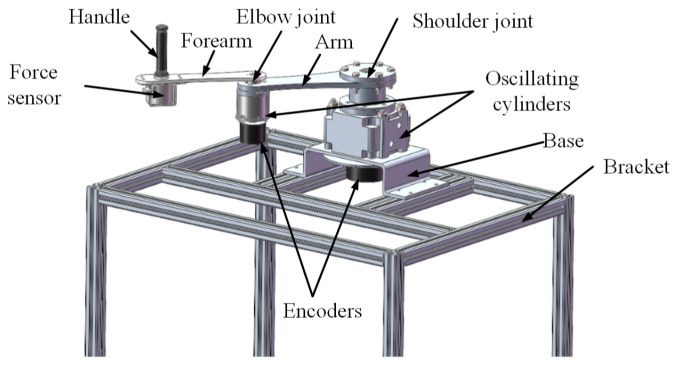
Three-dimensional structure of the upper-limb rehabilitation robot.

**Figure 2:**
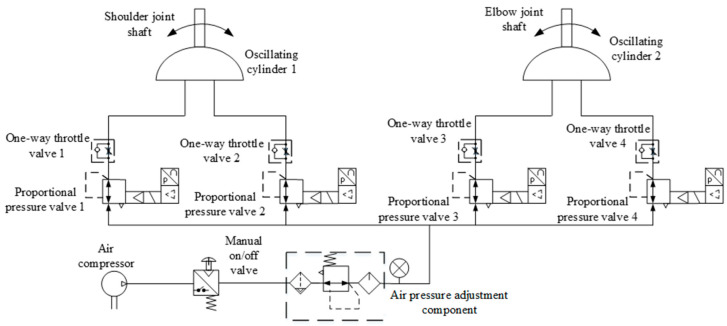
Pneumatic drive system for the robot joints.

The upper-limb rehabilitation training robot developed by us is a pneumatically driven end-effector robot which pulls the patient allowing for the completion of the rehabilitation exercise after grasping a handle. This robot is composed of an arm linkage, a forearm linkage, two joint components, a handle, and a base. The three-dimensional structure of the robot is shown in Figure 1, and the mechanical structural parameters of the robot are shown in Table 1. Differing from common systems in which servo motors drive the joints through a reducer, the robot’s joints are directly driven by oscillating cylinders, possessing reverse driving capability and exhibiting a certain level of compliance due to pneumatic driving. Each joint axis is equipped with incremental encoders to detect the joints’ rotation angles, while a three-dimensional force sensor is mounted at the end handle of the robot to measure human–robot interaction forces. Real-time detection by both sensors enables the collection and feedback of motion and force information. A joint oscillating cylinder is controlled by a pair of proportional pressure valves, with one side providing the driving force, and the other side providing back pressure to enhance start-up steadiness and motion stability. The pneumatic drive system for the robot joints is illustrated in Figure 2. By adjusting the control voltage of the proportional pressure valves through a control strategy, the swing angle and output torque of the oscillating cylinder can be modified to facilitate trajectory tracking control for rehabilitation and the dynamic adjustment of assisting forces. Proportional pressure valves, a gas supply system, and controllers are discreetly installed beneath the base bracket.

## 3. Personalized Assist-As-Needed Control Strategy

An adaptive AAN control strategy is proposed in this research. With this control strategy, the robot adjusts the assistance needs of the affected limb by comparing its capabilities with the movement characteristics of the healthy limb.

**Figure 3:**
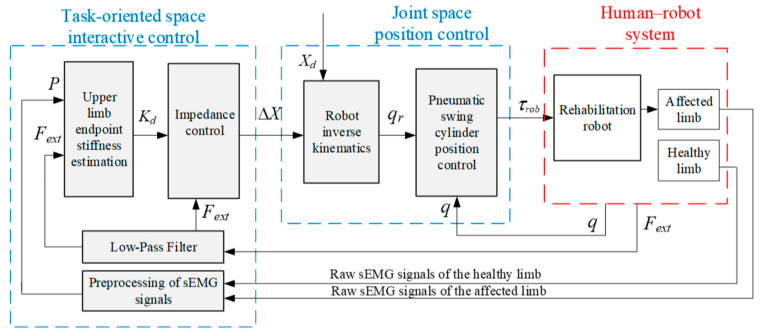
Block diagram of the AAN control system.

The end-effector robot interacts physically with the patient’s hand; so, rehabilitation tasks are planned in the task-oriented space, and the robot is controlled in the joint space. As shown in Figure 3, the strategy comprises an interactive control module in the task-oriented space based on the quantitative evaluation of the motion needs and an inner-loop position control module for the pneumatic swing cylinder in the joint space. In the position control module, the planned trajectory *X*_d_ and the adjusted value Δ*X* are converted into the desired joint angle *q*_r_ through inverse kinematics, and the robot joints angles *q* are controlled according to *q*_r_ through a position controller. A variable impedance control strategy based on patient upper-limb endpoint stiffness regulation was constructed in the interaction control module of the outer loop. With this strategy, the endpoint stiffness of the healthy limb is estimated based on the vector *P* of muscle activity obtained from preprocessed sEMG signals and then quantified as the stiffness value *K*_d_ of the impedance controller by comparison with the motor performance of the affected limb. Then, the task trajectory is adjusted according to the human–robot contact force *F*_ext_ between the affected limb and the robot, aiming to provide AAN rehabilitation motion. The AAN assessment is grounded in the stiffness characteristics of the patient’s own healthy limb when performing the interaction task. It better aligns with the force/position dynamic adjustment characteristics exhibited during the patient’s daily motions, thereby providing individualized advantages during the rehabilitation exercises.

### 3.1. Interactive Control Algorithms and Stiffness-Mapping Criteria

The impedance control strategy is a compelling method for effectively facilitating the robot–environment interaction, employing an impedance model to articulate the dynamic relationship between force and position [16,17]. On the basis of the inner-loop position control, the outer loop employs a position-based impedance control strategy for interactive control, as illustrated in Figure 3.

The impedance model of the robot in Cartesian space (i.e., the task-oriented space) is equivalent to a second-order dynamical system [18]:

(1) $$F_{\mathrm{ext}}=M_{d}\Delta \ddot{X}+B_{d}\Delta \dot{X}+K_{d}\Delta X$$

where $M_{d}$, $B_{d}$ and $K_{d}$ are the mass, damping, and stiffness coefficient matrix, respectively; ΔX is the position correction in the task-oriented space; $F_{\mathrm{ext}}$ is the external force applied to the robot.

The variation of the impedance parameters has varying degrees of impact on the effectiveness of robots in completing tasks [19]. In order to facilitate an effective rehabilitation process, robotic systems should exploit the patient’s physical capabilities and offer a proper stiffness range to provide assistance as needed in training processes. Consequently, an impedance control strategy with variable parameters is proposed in this study. This strategy considers the stiffness of the healthy limb and the affected limb as a reference and can dynamically adjust the impedance control parameters according to the patient’s movement performance, so that the patient’s rehabilitation needs can be met, and rehabilitation effectiveness can be enhanced.

The impedance parameters of the human upper limb are mapped to the impedance controller by designing a mapping criterion. As a result, the rehabilitation training robot, which mimics the motion characteristics of the patient’s healthy limb, drives the affected limb. This alignment ensures that the rehabilitation training motion is more consistent with the individual’s force generation habit. The parameter mapping criteria are outlined as follows:

(2) $$\left\{ \begin{array}{l} K_{d}=K_{\mathrm{endh}}-K_{\mathrm{enda}}\\ B_{d}=2\varepsilon \sqrt{K_{d}M_{d}} \end{array}\right.$$

where $K_{\mathrm{endh}}$ is the endpoint stiffness of the healthy limb for normal motion, estimated based on sEMG; $K_{\mathrm{enda}}$ is the endpoint stiffness of the affected limb for the same motion, estimated based on sEMG; ε is the damping ratio, which was selected to be 0.8 according to the stability requirements in pneumatic robots.

The interactive force between the affected limb and the robot adjusts the end-effector trajectory. The corrected trajectory is:

(3) $$X_{r}=X_{d}-\Delta X$$

where $X_{d}$ is the desired rehabilitation motion trajectory.

The robot joints are actuated by the swing cylinders, which need to be controlled in the joint space. As a consequence, the external torque $\tau_{\mathrm{ext}}$ can be obtained from the end interaction force $F_{\mathrm{ext}}$ using the force Jacobian matrix $J^{T}(q)$:

(4) $$\tau_{\mathrm{ext}}=J^{T}(q)F_{\mathrm{ext}}$$

The force Jacobian matrix $J^{T}(q)$ of the two-degree-of-freedom robot is represented as follows:

(5) $$J^{T}(q)=\left[\begin{array}{cc} -L_{1}\sin\theta_{1}-L_{2}\sin(\theta_{1}+\theta_{2}) & L_{1}\cos\theta_{1}+L_{2}\cos(\theta_{1}+\theta_{2})\\ -L_{2}\sin(\theta_{1}+\theta_{2}) & L_{2}\cos(\theta_{1}+\theta_{2}) \end{array}\right]$$

where $\theta_{1}$ and $\theta_{2}$ denote the joint angles of the upper arm and forearm, respectively, and *L*_1_ and *L*_2_ represent the lengths of the linkage segments corresponding to the robot’s upper arm and forearm.

Neglecting the influence of friction, the dynamical model of the robot in the joint space can be written as follow:

(6) $$M(\theta )\ddot{\theta }+C(\theta ,\dot{\theta })\dot{\theta }+\frac{1}{2}m_{1}gL_{1}+\frac{1}{2}m_{2}gL_{2}=\tau_{\mathrm{rob}}+\tau_{\mathrm{ext}}$$

with $\tau_{\mathrm{rob}}$ denoting the joint torque vectors output by the swing cylinder.

For the two-link planar robot shown in Figure 1, the dynamical equation coefficients in Equation (6) are described below.

The mass matrix is composed of all those terms which multiply $\ddot{\theta }$ and is a function of θ. Therefore, we have

$$M(\theta )=\left[\begin{array}{cc} M_{11} & M_{12}\\ M_{21} & M_{22} \end{array}\right]$$

with

(7) $$\left\{ \begin{array}{l} M_{11}=\frac{1}{3}{m_{1}L_{1}}^{2}+\frac{1}{3}{m_{2}L_{2}}^{2}+{m_{2}L_{1}}^{2}+m_{1}L_{1}L_{2}\cos\theta_{2}\\ M_{12}=\frac{1}{4}{m_{2}L_{2}}^{2}+\frac{1}{2}m_{2}L_{1}L_{2}\cos\theta_{2}\\ M_{21}=\frac{1}{4}{m_{2}L_{2}}^{2}+\frac{1}{2}m_{2}L_{1}L_{2}\cos\theta_{2}\\ M_{22}=\frac{1}{3}{m_{2}L_{2}}^{2} \end{array}\right.$$

The velocity term Coriolis/centrifugal matrix contains all those terms that have any dependence on joint velocity. Thus, we obtain

$$C(\theta ,\dot{\theta })=\left[\begin{array}{cc} C_{11} & C_{12}\\ C_{21} & C_{22} \end{array}\right]$$

with

(8) $$\left\{ \begin{array}{l} C_{11}=-{\dot{\theta }}_{2}m_{2}L_{1}L_{2}\sin\theta_{2}\\ C_{12}=-\frac{1}{2}m_{2}L_{1}L_{2}\sin(\theta_{2}){\dot{\theta }}_{2}\\ C_{21}=\frac{1}{2}m_{2}L_{1}L_{2}\sin(\theta_{2}){\dot{\theta }}_{1}\\ C_{22}=0 \end{array}\right.$$

In Equations (7) and (8), *m*_1_ and *m*_2_ are the concentrated masses of the robot’s upper arm and forearm.

### 3.2. Position Control Algorithms in the Joint Space

**Figure 4:**
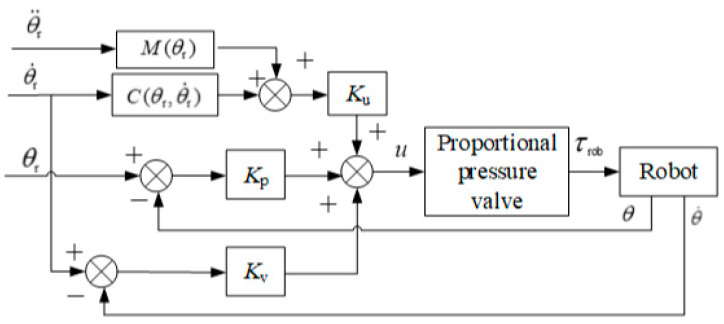
Position control strategy in the joint space.

The position control module employs the PD (proportional–derivative) control strategy with dynamic-term feedforward. To address the characteristics of the pneumatic proportional system [20], PD control was employed to increase system damping, thereby enhancing the system’s stability. Given the low-speed crawling issue in the swing cylinder and the joint motion coupling characteristics of the robot, dynamic-term feedforward control was introduced to compensate for torque, thereby improving the dynamic response of the pneumatic system and enhancing the robot’s position control accuracy. The schematic diagram of the position control strategy is shown in Figure 4.

The PD control with the dynamic-term feedforward algorithm is outlined as follows:

(9) $$\left\{ \begin{array}{l} u=u_{1}+K_{u}\left(M(\theta_{r}){\ddot{\theta }}_{r}+C(\theta_{r},{\dot{\theta }}_{r})\right)\\ u_{1}=K_{p}(\theta_{r}-\theta )+K_{v}({\dot{\theta }}_{r}-\dot{\theta }) \end{array}\right.$$

where $\theta_{r}$ is the desired joint trajectory; θ is the actual joint trajectory; $u_{1}$ is the output voltage of the PD control; u is the control voltage for the proportional pressure valve; $K_{u}$ is the proportional coefficient matrix of the dynamic-term feedforward control; and $K_{p}$ and $K_{V}$ are the proportional and differential coefficient matrices of the PD control, respectively.

The control voltage of the proportional pressure valve is linearly related to the output torque of the swing cylinder installed on the robot to drive the joints. The output torque $\tau_{\mathrm{rob}}$ is calculated by Equation (10), and the proportional coefficient *K*_τ_ can be determined experimentally

(10) $$\tau_{\mathrm{rob}}=K_{\tau }u$$

## 4. Human Arm Endpoint Stiffness Estimation Method

The mechanical characteristics of the human arm reflect a patient’s movement ability in the motion interaction of the end-effector rehabilitation robot dragging the patient. One way to quantify the interaction between the limb and the robot in rehabilitation tasks is through the estimation of the endpoint stiffness. The stiffness can be modified via co-contraction of the muscles involved in task execution [21]. Therefore, an upper-limb endpoint stiffness estimation model based on sEMG signals was constructed to map muscular activities and the resulting arm endpoint force and stiffness. Then, the parameters of the model were determined by the small perturbation method.

### 4.1. Endpoint Stiffness Estimation Modeling

When humans perform tasks, the force required for task completion is generated by altering the activation pattern of individual muscles, and the stiffness for task completion is regulated through the co-contraction of muscle groups, with both processes operating independently. Specifically, the resulting modifications in force and impedance can be regarded as the effects of internal force regulation exerted by the extensor and flexor muscles. Agonist–antagonist muscle co-contractions affect and modify the endpoint stiffness of the arm. In the equilibrium position, the counterbalance of flexor and extensor muscle forces results in no force variation and joint rotation, but the co-contraction of the muscles leads to an increase in stiffness. When maintaining posture amidst mechanical perturbation, the changes in force and impedance exhibit a linear relationship with the level of muscle activation [22,23]. The mapping of muscular activities and resulting arm endpoint force and stiffness in Cartesian coordinates was described in [24]

(11) $$\left[\begin{array}{c} F_{\mathrm{end}}\\ K_{\mathrm{end}} \end{array}\right]=\left[\begin{array}{c} T_{F}\\ T_{K} \end{array}\right]P+\left[\begin{array}{c} 0\\ K_{0} \end{array}\right]$$

where $F_{\mathrm{end}}$ and $K_{\mathrm{end}}$ represent the endpoint force and stiffness vectors, respectively; $T_{F}$ is the EMG-to-force map matrix; $T_{K}$ is the EMG-to-stiffness map matrix; *P* is the vector of muscular activities, obtained from preprocessing EMG signals from electrodes applied on each muscle; and *K*_0_ is the intrinsic stiffness in relaxed conditions.

The robot pulls the patient producing movement in the plane, and thus both $F_{\mathrm{end}}$ and $K_{\mathrm{end}}$ are two-dimensional vectors. The EMG-to-force map matrix $T_{F}$ is defined as

(12) $$T_{F}=\left[\begin{array}{cccccc} \alpha_{x1} & \cdots & \alpha_{xi} & \beta_{x1} & \cdots & \beta_{xi}\\ \alpha_{y1} & \cdots & \alpha_{yi} & \beta_{y1} & \cdots & \beta_{yi} \end{array}\right]$$

where $\alpha_{xi}$ and $\alpha_{yi}$ are the force-map coefficients of the i-th agonist muscle in the X and Y directions, and $\beta_{xi}$ and $\beta_{yi}$ are the force-map coefficients of the i-th antagonist muscle in the X and Y directions.

The identification of the EMG-to-force map matrix $T_{F}$ is relatively easily performed by conducting precise measurements of the endpoint force and the EMG signals of the individual muscles, while it is more challenging to identify the EMG-to-stiffness map matrix $T_{K}$ by the EMG signals due to the co-contraction of muscle groups. To solve this problem, Arash Ajoudani [21] proposed an algorithm for estimating human arm stiffness using the force-map null space $P_{k}$ which contains information about the co-contraction component of stiffness generation.

The space of muscular activation *P* is the direct sum of a force-generating subspace $P_{F}$ and the force-map null space $P_{k}$, i.e.,

(13) $$P=P_{F}⊕P_{k}$$

*N*_F_ denotes a basis matrix for the kernel of *T*_F_, written as

(14) $$N_{F}=I-T_{F}^{R}T_{F}$$

where $T_{F}^{R}$ is the right-inverse matrix of *T*_F_, i.e., $T_{F}T_{F}^{R}=I$.

Consequently, the null-space component $P_{k}$ can be expressed as

(15) $$P_{k}=N_{F}P$$

The model for Cartesian stiffness regulation through co-contraction is formulated as

(16) $$K_{\mathrm{end}}=K_{\mathrm{end}0}+T_{c}P_{k}$$

where $T_{c}$ maps the force-map null space $P_{k}$ (the set of muscle activations that do not change the endpoint force) in relation to stiffness variations.

From the above algorithms, it can be seen that the established estimation model for endpoint stiffness based on force-map null space vectors describes the relationship between muscle co-contraction and stiffness.

### 4.2. Parameter Identification in the Stiffness Estimation Model

#### 4.2.1. Identification of the EMG-To-Force Map Matrix $T_{F}$

The relationship between the endpoint force and the muscular activity vector can be derived from Equation (11) as

(17) $$F_{\mathrm{end}}=T_{F}P$$

The dataset {$F_{\mathrm{end}}$, *P*} is constructed by acquiring multiple sets of EMG signals from dominant muscles and the endpoint forces during the completion of rehabilitation training tasks. Then, the identification of the EMG-to-force map matrix $T_{F}$ can be accomplished by utilizing the projected gradient descent algorithm for the linear Equation (17).

For the end-effector rehabilitation robot, dragging the patient’s upper limb in plane to accomplish rehabilitation, we estimated $T_{F}$ by combining the endpoint force vectors in the horizontal plane with the measured activities of four involved muscles. The endpoint forces in the horizontal plane, $F_{\mathrm{end}}$= [$F_{x}$ $F_{y}$]^T^, were detected by a three-axis force sensor connected to a handle equipped at the endpoint of the robot arm. The analogue sEMG signals from the dominant muscles associated with the shoulder and elbow joint motions were collected and amplified separately utilizing an EMG signal detector. Four dominant muscles acting on elbow and shoulder joints [25] were chosen as the sources of sEMG recordings, including two flexors, i.e., the biceps long head (BILH) and the deltoid clavicular part (DELC), and two extensors, i.e., the triceps lateral head (TRIA) and the deltoid scapular part (DELS). Since the raw surface electromyographic (sEMG) signals directly acquired through electrode pads contain information about the activity of motor units of muscle fibers, along with some noise signals, a preprocessing algorithm was employed to extract the envelope amplitude of the raw sEMG signals. The preprocessing algorithm included procedures such as linear noise removal, Butterworth bandpass filtering, and root-mean-square envelope calculation.

#### 4.2.2. Identification of the EMG-to-Stiffness Map Matrix $T_{c}$

From Equations (14)–(16), it can be inferred that the kernel matrix *N*_F_ was initially obtained from the identified EMG-to-force map matrix $T_{F}$. Subsequently, the identification of the EMG-to-stiffness map matrix $T_{c}$ for the linear Equation (16) was achieved through the application of the projection gradient descent algorithm.

During the identification process, the participant grasps the handle at the robot’s end-effector and generates random perturbation forces of a certain peak value in the X and Y directions by varying the degree of muscle contraction, thereby creating a sufficient dataset. This interactive process is described by the Cartesian space impedance model:

(18) $$F_{H}=M_{\mathrm{end}}\ddot{X}+B_{\mathrm{end}}\dot{X}+K_{\mathrm{end}}(X-X_{0})$$

where $F_{H}$ is the human–robot interaction force, $M_{\mathrm{end}}$, $B_{\mathrm{end}}$, and $K_{\mathrm{end}}$ are the mass, damping, and stiffness matrices of the endpoint of the upper limb, respectively; X is the endpoint position; and $X_{0}$ is the initial-point position.

During the experiment, it is necessary for the testers to perform contraction motions at varying levels of muscle activity. This can be monitored using the co-contraction index *TCI* [25], defined as follows:

(19) $$TCI=S_{xx}+S_{yy}$$

where $S_{xx}$ and $S_{yy}$ are the contraction indices corresponding to the forces acting in the X and Y directions at the end-effector in a plane. They can be determined by the identified EMG-to-force map matrix $T_{F}$ and the muscular activity vector *P*:

(20) $$\left[\begin{array}{c} S_{xx}\\ S_{yy} \end{array}\right]=\left|T_{F}\right|P$$

The co-contraction index (*TCI*) needs to be normalized for different patients, with *TCI*_min_ representing muscle relaxation, and *TCI*_max_ representing full muscle contraction. The muscle contraction rate $\psi_{\mathrm{co}}$ is defined based on the normalized *TCI* index:

(21) $$\psi_{\mathrm{co}}=\frac{TCI-TCI_{\min}}{TCI_{\max}-TCI_{\min}}$$

When the muscles are in a relaxed state, $\psi_{\mathrm{co}}$ = 0, as indicated by Equation (18). By applying a small mechanical perturbation to the endpoint of the human arm by the robot, the initial intrinsic impedance parameters ($M_{\mathrm{end}0}$, $B_{\mathrm{end}0}$, $K_{\mathrm{end}0}$) can be obtained based on the measured restoring force and position deviation. When the patient performs active motions, the muscles are in a certain contraction state, exerting force on the robot. The endpoint stiffness of the human upper limb $K_{\mathrm{end}}$ is calculated by Equation (18) at this time. Let $K_{e}=K_{\mathrm{end}}-K_{\mathrm{end}0}$, then the Equation (16) is rewritten as

(22) $$K_{e}=T_{c}P_{k}$$

For the linear Equation (22), the endpoint-stiffness mapping matrix $T_{c}$ identification can also be accomplished by employing the projected gradient descent algorithm.

## 5. Experiments and Results Analysis

**Figure 5:**
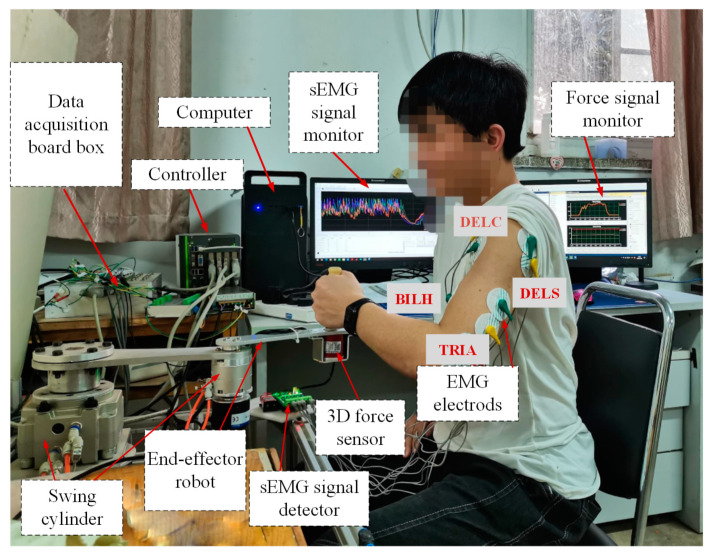
Physical prototype of the robot and the test system.

In order to evaluate the effectiveness of the proposed control method, experiments were carried out. The experimental system primarily consisted of a two-degree-of-freedom end-effector rehabilitation training robot prototype driven by pneumatic swing cylinders, a computer, a controller, data acquisition boards, a three-dimensional force sensor, and an sEMG signal acquisition device, as depicted in Figure 5.

The pneumatic swing cylinders were selected from the products of SMC Corporation (Tokyo, Japan), with the CRB1BW100-270S model used for the upper arm and the CRB2BW40-270S model for the forearm. The cylinder control valves were the VPPE-3-1-1/8-6-010-E1 proportional pressure valves produced by FESTO Corporation (Esslingen, Germany). The control system of the robot adopted the Links-RT semi-physical simulation device, with control board cards embedded internally. Specifically, the PCI-6602 board card (National Instruments (NI), Austin, TX, USA) was utilized for collecting pulse signals output by the encoders, the PCI-6251 board card (NI) served as an A/D input card for acquiring the output pressure of proportional pressure valves and the human–robot interaction force feedback from the force sensors, while the PCI-6216 board card (NI) functioned as an output card, generating analog voltage signals to control the pressure of the proportional pressure valves. Within the sensing system, two incremental encoders were selected for robot joint angle acquisition. The A3D46 model (Shenzhen Measurement and Control Technology Co., Ltd, Shenzhen, China) three-dimensional force sensor was chosen for human–robot interaction force acquisition, operating in the range from 0 to 200 N in the XYZ directions, while the pressure sensor selected for proportional valve output pressure acquisition was the PSE540-R06 series produced by SMC Corporation, with a rated pressure range from 0 to 1 MPa. Additionally, a six-channel sEMG sensor developed by SICHIRAY Corporation (Wuxi, China) was utilized for collecting human raw sEMG signals.

### 5.1. Parameter Identification and Stiffness Estimation Experiment

**Figure 6:**
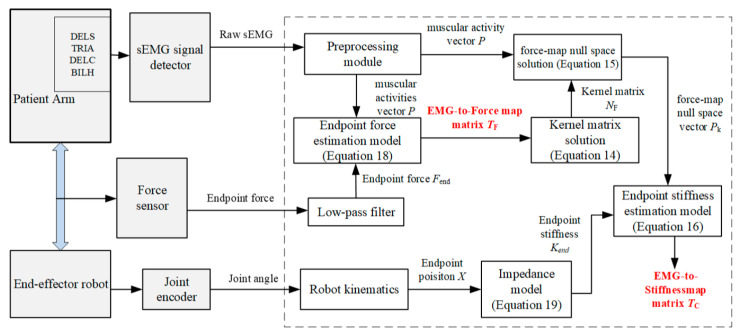
Experimental and computational procedure for endpoint stiffness identification based on sEMG signals.

Based on the stiffness estimation model, the experimental and computational procedures were designed according to the parameter identification method explained in Section 4, as illustrated in Figure 6.

Initially, the identification of the EMG-to-force map matrix $T_{F}$ was completed. The robot was in a no-drive mode and served only as a support for the force sensors. The test subject sat upright, naturally positioning the upper arm and tightly gripping the robot handle by the hand. The participant, with electrode sheets affixed to four muscles of the healthy limb, generated a specified force in the X and Y directions. Six sets of force data and twelve sets of sEMG data (across four channels) were obtained by measuring three times in each direction. Using these data, the identification of $T_{F}$ was achieved through the application of the projected gradient descent algorithm, and the results were as follows.

$$T_{F}=\left[\begin{array}{cccc} 0.306 & 0.152 & -0.185 & 0.651\\ 0.876 & -0.319 & 0.583 & -0.582 \end{array}\right]$$

Based on the identified $T_{F}$, the kernel matrix $N_{F}$ was calculated by Equation (14).

$$N_{F}=\left[\begin{array}{cccc} 0.1088 & 1.0967 & 0.7430 & 0.8533\\ 1.0967 & 0.9165 & 1.1356 & 0.7791\\ 0.7430 & 1.1356 & 0.7669 & 1.3071\\ 0.8533 & 0.7791 & 1.3071 & 0.2078 \end{array}\right]$$

**Figure 7:**
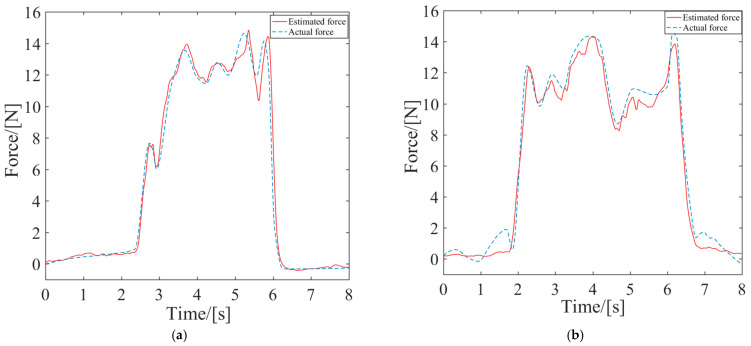
Comparison of actual force and estimated force in the X and Y directions. (**a**) Actual and estimated force in the X-direction; (**b**) Actual and estimated force in the Y-direction.

The estimated force was calculated by Equation (17) and was then compared to the actual force measured by the force sensor, as depicted by the curve in Figure 7. Consequently, the validity of proposed identification method was demonstrated, providing a good representation of the mapping relationship between the sEMG signals and endpoint forces.

In the following phase, the upper-limb end-effector rehabilitation robot transitioned to an active control mode. The participant gripped the handle at the robot’s endpoint, generating random perturbations with specific peaks in the X and Y directions. The intrinsic impedance parameters ($M_{\mathrm{end}0}$, $B_{\mathrm{end}0}$, $K_{\mathrm{end}0}$) of the upper-limb endpoint were calculated by Equation (18) in a relaxed state of the muscles in the healthy limb, specifically, at a contraction rate $\psi_{\mathrm{co}}$ = 0. These parameters are detailed in Table 2.

The human–robot interaction force and positional deviation were detected again during muscle contraction of the healthy limb, and the endpoint stiffness $K_{\mathrm{end}}$ of the upper limb was calculated by Equation (18). Finally, the endpoint stiffness mapping matrix was identified, and the results are as follows.

$$T_{c}=\left[\begin{array}{cccc} 293.8 & 494.4 & -217.8 & -390.1\\ 327.2 & 583.5 & -353.1 & -496.3 \end{array}\right]$$

### 5.2. Assist-As-Needed Control Experiment

Within the safe working space of the experimental prototype, rehabilitation training experiments with AAN control were conducted by planning a circular motion trajectory in Cartesian space. Multiple healthy individuals participated in the experiments, simulating different degrees of motion impairments. The robot pulled their arms to perform circular training in the plane.

**Figure 8:**
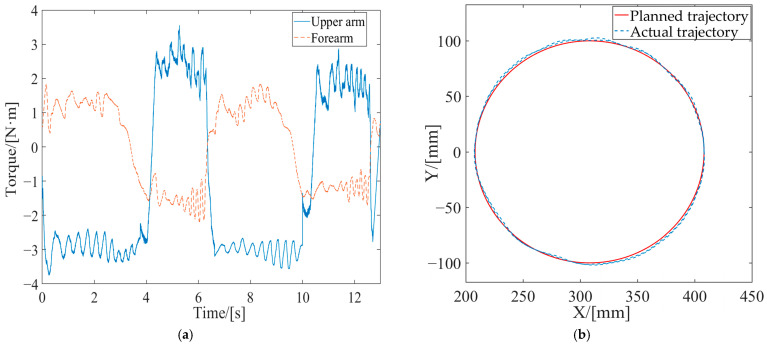
Experimental curves of passive training under position control. (**a**) Joint torque; (**b**) endpoint motion trajectory.

Firstly, the effectiveness of the inner-loop position controller was verified through passive rehabilitation training experiments. The passive rehabilitation training mode refers to training that was completely driven by robots when the affected limb was in a flaccid state. Due to the lack of a human–robot contact force during this training process, the interaction control of the outer loop was not effective, and the robot joint motion was only controlled by the position controller to track the planned circular training trajectory. The robot’s joints were driven by the swing cylinders, which were controlled by the proportional pressure valves. Precision in pneumatic joints’ position control was ensured through the PD control with the dynamic-term feedforward strategy, as mentioned in Section 3. As shown in Figure 2, each swinging cylinder of the driving joint is equipped with two proportional pressure valves, which realize the movement of the swinging cylinder based on the pressure difference. The output torque of the swinging cylinder is linearly proportional to the control voltage difference of the two proportional pressure-regulating valves. With the pressure sensors and an A/D (analog/digital) board, the output pressure and analog voltage changes related to the proportional valves were measured. Using the torque and pressure difference coefficients provided in the swing cylinder manual, the coefficient *K*_τ_ was calculated. The stable pressure provided by the pump during operation was 0.6 MPa, and the coefficient *K*_τ_ of the proportional pressure valve was experimentally determined to be 0.06. The control parameters are listed in Table 3. The motion trajectory at the robot’s endpoint and the joint torque curves are depicted in Figure 8.

During passive rehabilitation training, the robot provided the patient with the maximum assistive torque, given that the affected limb had no active participation ability. As shown in Figure 8a, the joint torque provided by the robot upper arm was about 3 Nm, and the joint torque provided by the forearm was about 1.5 Nm. At the same time, the robot drove the affected limb according to the desired trajectory with a small error, as shown in Figure 8b. With this system, the desired trajectory is planned according to the bearing capacity of the affected limb. For example, if the range of motion of the affected limb is small, the radius of the circle in early rehabilitation training will be set accordingly, and the training trajectory will gradually increase with the degree of rehabilitation of the affected limb. The average absolute position errors in the *X*-axis and *Y*-axis relative to the desired trajectory were 2.13 mm and 3.05 mm, respectively, through statistical calculations. The position accuracy met the needs of rehabilitation training, thus verifying the effectiveness of the design of the position control strategy.

**Figure 9:**
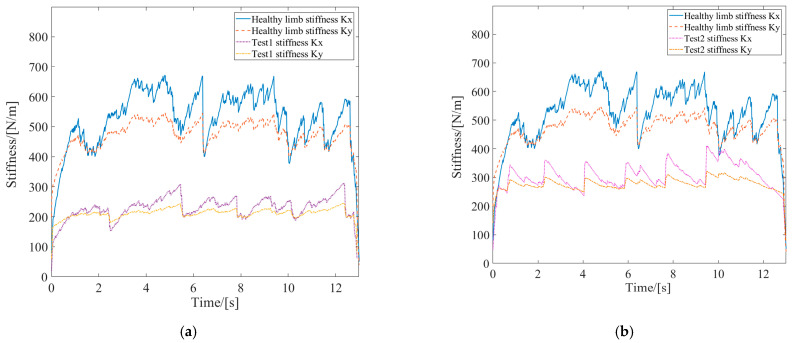
Stiffness curves of healthy limb and affected limb in active rehabilitation training. (**a**) Test 1; (**b**) Test 2.

**Figure 10:**
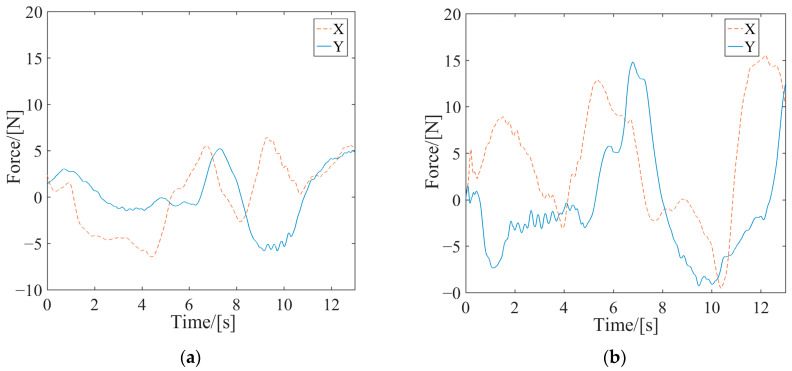
The curves of the human–computer interaction force in active rehabilitation training. (**a**) Test 1; (**b**) Test 2.

**Figure 11:**
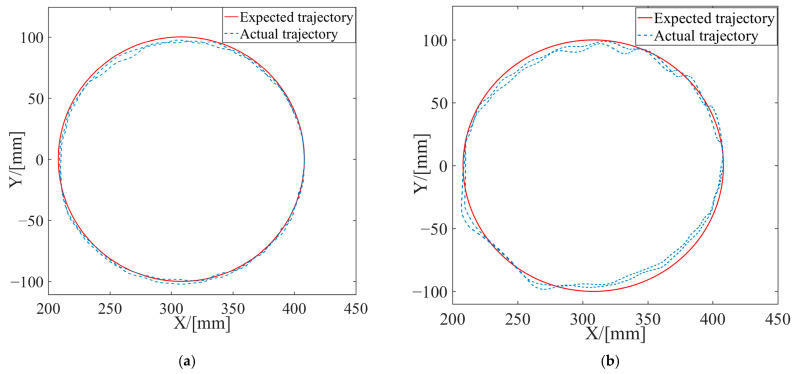
The end trajectories of the robot in active rehabilitation training. (**a**) Test 1; (**b**) Test 2.

**Figure 12:**
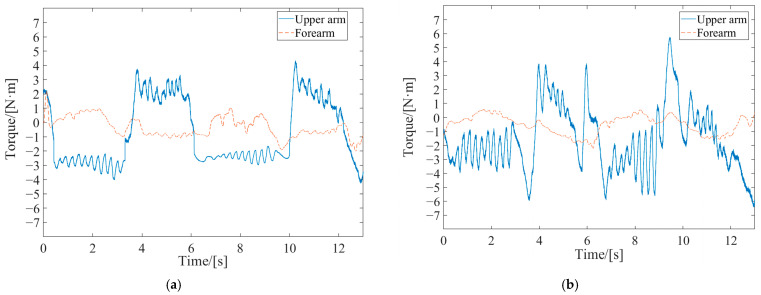
Robot joint torques in active rehabilitation training. (**a**) Test 1; (**b**) Test 2.

Active rehabilitation training is employed when the affected limb possesses a certain level of motion capability and was performed utilizing the AAN control strategy proposed in this study. In the AAN control experiments, the tester interacted with the robot by varying muscle contraction intensity and output force to imitate affected limbs with different motor abilities. These were referred to as Test 1 and Test 2 and labeled in the experimental curves. EMG signals from both the healthy and the affected limbs were collected. Then, the estimated stiffnesses were obtained by utilizing the stiffness estimation algorithm, and the curves are shown in Figure 9. The forces exerted by the affected limb on the robot were synchronously acquired, as depicted in Figure 10. The robot endpoint motion trajectory under AAN control is illustrated in Figure 11, while the robot joint torques are presented in Figure 12.

## 6. Discussion

Upon examining the aforementioned test data in Figure 9, Figure 10, Figure 11 and Figure 12, the AAN characteristics of the robot in active rehabilitation training were analyzed and are discussed in detail below.

(1) As shown in Figure 9, the mean value of the endpoint stiffness in the X-direction of the healthy limb was approximately 550 N/m, and the mean value of the endpoint stiffness in the Y-direction was about 450 N/m. The endpoint stiffness values in both Test 1 and Test 2 were lower than those of the healthy limb, with Test 1 showing a lower stiffness than Test 2. Therefore, Test 1 was used to simulate the more severely affected limb, while Test 2 was used to simulate the less severely affected limb. The testers could not entirely maintain the same muscle contraction intensity during motion; so, the measured stiffness had the characteristic of fluctuating at a constant level. Meanwhile, the stiffness in the X-direction was slightly greater than that in the Y-direction when completing the task of drawing a circle. This observation reflects the stiffness characteristics of human motion in different directions during the task, indicating good consistency in the algorithm’s recognition.

(2) The real-time changes in endpoint forces detected by the force sensor mounted at the handle’s endpoint in the two tests are illustrated in Figure 10. A comparison revealed that the variation trends of the endpoint forces in the two experiments were similar. Nevertheless, the endpoint force in Test 1 was significantly lower than that in Test 2. Corresponding to the stiffness curves in Figure 9, since the affected-limb condition simulated in Test 1 was more serious than that in Test 2, the force curves indicated that the patient in Test 2 had a greater active motion ability.

(3) The variations between the expected and the actual endpoint motion trajectories of the robot during the two tests are depicted in Figure 11. Firstly, compared to the endpoint motion trajectory in passive rehabilitation training (Figure 8a), the desired trajectory was regulated by the impedance model because of the addition of the interaction control outer loop and the participation of the affected limb in active rehabilitation training. The robot moved according to the adjusted trajectory, demonstrating its motion compliance. Comparing Figure 12a,b, the amount of trajectory adjustment in Figure 12b is greater due to the higher active involvement of the affected limb in Test 2.

(4) The output joint torque reflects the robot’s AAN characteristics, which can be calculated by Equation (10) with the air pressure values detected in the two chambers of the swing cylinder. Comparing the joint torque in passive training (Figure 8b), the average assistive torques provided by the robot’s upper arm and forearm in Test 1 in Figure 12a and Test 2 in Figure 12b were reduced by 0.68 Nm and 0.212 Nm, respectively. This suggests that the robot provided varying assistance based on the participant’s motion performance.

Tatsuya Teramae et al. [11] also proposed an EMG-based assist-as-needed (AAN) controller for rehabilitation. The joint torque of a patient was estimated from the measured EMG signals of the affected limb, and then the deficient joint torque was derived based on the preset desired torque to generate the target movements. The adaptive AAN control focuses on assessing in a patient the motor functional ability of the affected limb only. As a result, the outcomes of the AAN control, in which the designer determines the desired outcome, tend to diverge from the patient’s expectations. In our study, the active movement ability of affected limbs in different conditions was quantitatively described through the endpoint stiffness. Subsequently, compared to the normal movement ability of healthy limbs, the varying assistive forces provided by the robot were determined. This variation reflects personalized assist-as-needed features tailored to the patient’s active movement abilities. Furthermore, the stiffness directionality of a patient during task completion is also integrated into the auxiliary strategy due to the different stiffness values mapped to the robot in the X and Y directions. This integration highlights the second advantage of this method—the incorporation of robotic assistive features tailored to the patient’s unique arm exertion habit.

## 7. Conclusions

In this paper, an AAN control method based on the motion characteristics of the healthy limb is proposed, using a pneumatic end-effector upper-limb rehabilitation training robot as the controlled device. To evaluate the patient’s motion performance, a stiffness estimation model was established by utilizing the sEMG signals containing skeletal muscle motion information, and a parameter identification method was designed to obtain the estimated endpoint stiffness of the patient’s arm. A stiffness mapping algorithm was developed for the impedance controller. The impedance parameters of the controller were synchronously adjusted in real time based on the estimated stiffnesses of the healthy and the affected limb. By using the stiffness information of the healthy limb as a benchmark for the required motion capability to complete tasks and combining it with that of the affected limb to assess the motor needs, the robot’s assistive force can be dynamically modified. This method employs personalized stiffness parameters representing the force/position dynamic relationship in human–robot interaction, so that the robot-assisted force for completing the rehabilitation task is better aligned with the patient’s pre-morbid limb habit, thereby promoting coordination in bilateral motions.

Through prototype experiments, including the estimation of the endpoint stiffness of the human arm based on sEMG, the position control of the robot, and variable impedance AAN control experiments, the results demonstrate that the established stiffness estimation model and identification algorithm provide a correct quantitative method for estimating the motion ability of a patient’s limb. The robotic assistance torque and motion trajectory undergo reasonable adaptive adjustments with changes in the motion ability of the affected limb and in human–robot interaction forces, thereby validating the effectiveness of the AAN control strategy proposed in this study and affirming the feasibility of the pneumatic robot system design. The proposed techniques in this study contribute to enhancing personal adaptation in robot-assisted active rehabilitation training.

Although the results obtained in this paper are encouraging, the algorithm for estimating the endpoint stiffness in three-dimensional motion needs further derivation. Among the limitations of the present technique is the local validity of the stiffness estimation data. This arises from the fact that, in the experiments described in this article, the upper limbs completed plane movements in a relatively consistent posture. When applied to other multi-degree-of-freedom rehabilitation training robots, such as those completing spatial movements, three-dimensional stiffness recognition is related to upper-limb posture and needs to be recalibrated in different postures. Additionally, the rehabilitation training robot is still in the prototype stage, and the selected experimenters were all healthy individuals. The system was validated by simulating patients. In the future, clinical trial studies will be carried out to further optimize the design of the system.

## Figures and Tables

**Table 1:** Robot parameters.

Name	Length/mm	Mass/Kg
Arm	228.15	0.76
Forearm	180	0.148

**Table 2:** Intrinsic impedance parameters of the healthy-limb endpoint.

$M_{0}$ (Kg)		$B_{0}$ (Ns/m)		$K_{0}$ (N/m)	
$M_{xx}$	$M_{yy}$	$B_{xx}$	$B_{yy}$	$K_{xx}$	$K_{yy}$
0.21	0.15	14.9	25.2	194.1	178.3

**Table 3:** Position control parameters.

	*K*_p_	*K*_d_	*K*_u_
Swing cylinder of the upper arm	0.23	0.024	0.012
Swing cylinder of the forearm	0.4	0.015	0.04

## Data Availability

All the test data mentioned in this paper will be made available upon request to the corresponding author with appropriate justification.
